# The Kynurenine Pathway in Gut Permeability and Inflammation

**DOI:** 10.1007/s10753-024-02135-x

**Published:** 2024-09-10

**Authors:** Rowan Kearns

**Affiliations:** Ulster University, Life and Health Sciences, Newry, Northern Ireland United Kingdom

**Keywords:** Kynurenine Pathway, Gut-brain Axis, Neuroinflammation, Probiotics, Gut Permeability

## Abstract

The gut-brain axis (GBA) is a crucial communication network linking the gastrointestinal (GI) tract and the central nervous system (CNS). The gut microbiota significantly influences metabolic, immune, and neural functions by generating a diverse array of bioactive compounds that modulate brain function and maintain homeostasis. A pivotal mechanism in this communication is the kynurenine pathway, which metabolises tryptophan into various derivatives, including neuroactive and neurotoxic compounds. Alterations in gut microbiota composition can increase gut permeability, triggering inflammation and neuroinflammation, and contributing to neuropsychiatric disorders. This review elucidates the mechanisms by which changes in gut permeability may lead to systemic inflammation and neuroinflammation, with a focus on the kynurenine pathway. We explore how probiotics can modulate the kynurenine pathway and reduce neuroinflammation, highlighting their potential as therapeutic interventions for neuropsychiatric disorders. The review integrates experimental data, discusses the balance between neurotoxic and neuroprotective kynurenine metabolites, and examines the role of probiotics in regulating inflammation, cognitive development, and gut-brain axis functions. The insights provided aim to guide future research and therapeutic strategies for mitigating GI complaints and their neurological consequences.

## Introduction

The gut-brain axis (GBA) is a critical communication network linking the gastrointestinal (GI) tract and the central nervous system (CNS). Acting as an anaerobic bioreactor, the gut microbiota significantly affects metabolic, immune, and neural functions by generating a diverse array of microbial metabolites, peptides, gut hormones, and neuroactive substances [15, 31]. These bioactive compounds travel via the ENS, circulatory system, vagus nerve, and immune system, thereby modulating brain function and contributing to the maintenance of homeostasis [5, 30, 64].

A key mechanism in gut-brain communication is the kynurenine pathway (KP), the main route for the metabolism of essential amino acid, tryptophan (TRP). Approximately 90% of dietary TRP is converted into kynurenine and its derivatives via this pathway, with the remainder metabolised into serotonin and indole compounds. The KP plays a significant role in the GBA and is involved in various physiological and pathological processes, including neuroinflammation and neurodegeneration. Alterations in gut microbiota composition and function can result in increased gut permeability, triggering inflammation and neuroinflammation. This process is implicated in the pathophysiology of numerous neuropsychiatric disorders [31, 64].

Advancements in high-throughput sequencing and metabolomic analysis have expanded our comprehension of the gut's role in systemic health, extending beyond its primary digestive and absorptive functions. It is now acknowledged that the GI tract plays a crucial role in immune regulation and systemic inflammation. Physiological stressors, such as reduced blood flow and pathogenic invasions, can disrupt GI homeostasis, leading to symptoms such as bloating, cramping, and diarrhoea [75], [117]. These GI dysfunctions can further impact the CNS via the GBA, affecting mood, cognitive functions, and stress responses.

This review elucidates the mechanisms by which changes in gut permeability may lead to systemic inflammation and subsequent neuroinflammation, focusing particularly on the KP. By delineating these mechanisms, we aim to provide insights into potential therapeutic strategies for mitigating GI complaints and their neurological consequences. Special attention will be given to the role of probiotics in modulating the KP and reducing neuroinflammation.

## Molecular Mechanisms of the Kynurenine Pathway

The kynurenine pathway (KP) serves as the primary route for the metabolism of TRP, an essential amino acid integral to numerous physiological functions. This pathway plays a significant role in the GBA and is involved in a variety of physiological and pathological processes, including neuroinflammation and neurodegeneration.

**Fig. 1:**
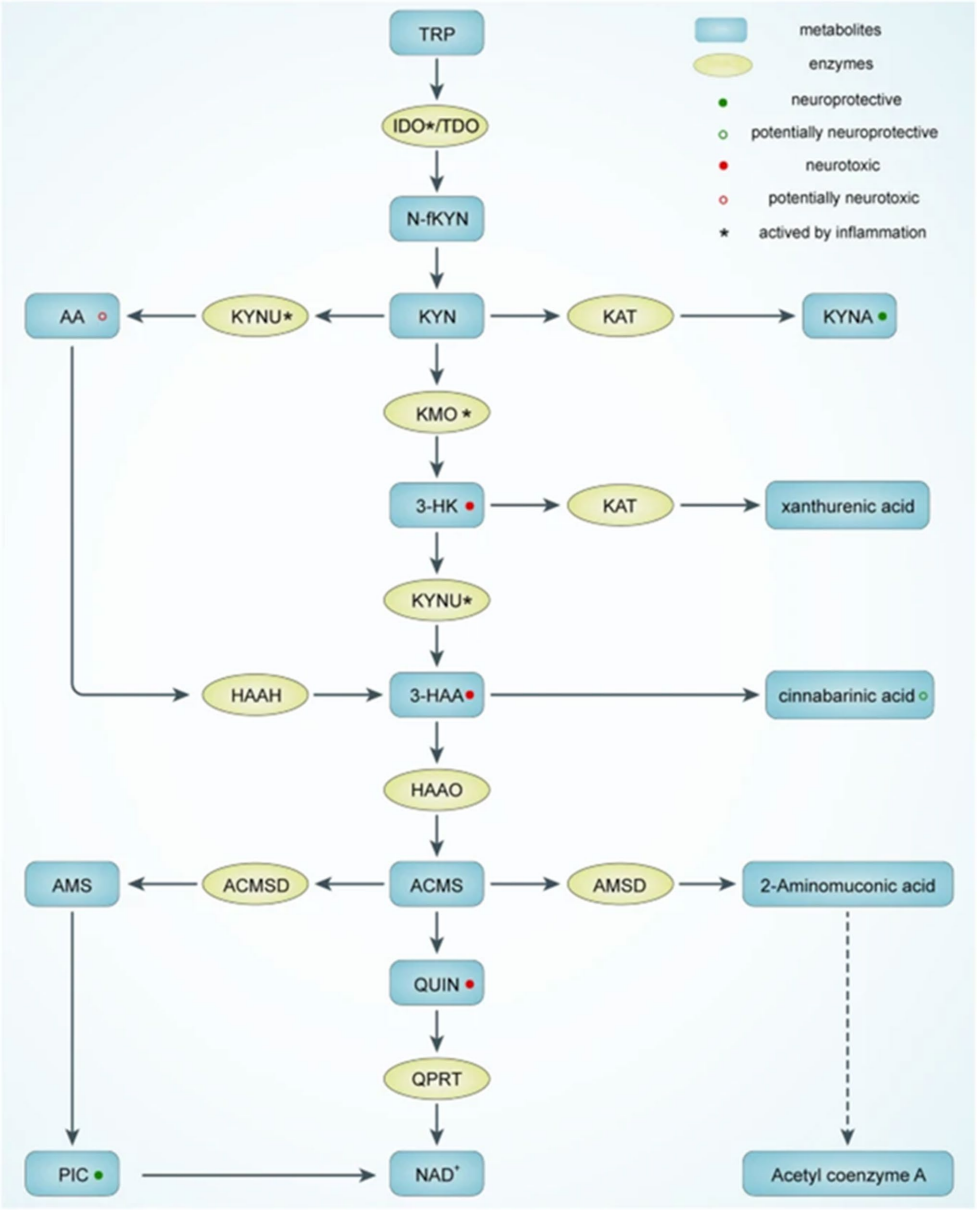
The tryptophan-kynurenine metabolic pathway.

The KP begins with the conversion of TRP to N-formylkynurenine (N-fKYN), a reaction catalysed by the rate-limiting enzymes indoleamine 2,3-dioxygenase (IDO) and tryptophan 2,3-dioxygenase (TDO). Subsequently, N-fKYN is hydrolysed to kynurenine (KYN) by kynurenine formamidase. TDO primarily facilitates the basal metabolism of TRP in the liver, whereas IDO is predominantly active in immune cells and can be induced by pro-inflammatory cytokines such as interferon-gamma (IFN-γ), interleukin-1 (IL-1), IL-6, and tumour necrosis factor-alpha (TNF-α)([21, 22, 52]) (Fig. 1).

KYN serves as a crucial metabolite within the pathway, branching into several important metabolites. The first branch involves the conversion of KYN into kynurenic acid (KYNA) via the enzyme kynurenine aminotransferase (KAT). KYNA is recognised for its neuroprotective properties, acting as an antagonist to the N-methyl-D-aspartate (NMDA) receptor. The second branch converts KYN into anthranilic acid (AA) through the action of kynureninase (KYNU). The third branch transforms KYN into 3-hydroxykynurenine (3-HK) via kynurenine 3-monooxygenase (KMO) [106].

3-HK can be further metabolised into 3-hydroxyanthranilic acid (3-HAA) by KYNU or into xanthurenic acid (XA) by KAT. In the brain, AA is efficiently converted into 3-HAA, which can subsequently form cinnabarinic acid (CA) or 2-amino-3-carboxymuconate-6-semialdehyde (ACMS). ACMS has several metabolic fates: it can be converted to quinolinic acid (QUIN), an NMDA receptor agonist and neurotoxin, or to picolinic acid (PIC) via 2-amino-3-carboxymuconate-6-semialdehyde decarboxylase (ACMSD). QUIN is particularly noteworthy as it can further metabolise into nicotinamide adenine dinucleotide (NAD +) via quinolinate phosphoribosyltransferase (QPRT), underscoring the pathway's role in cellular energy metabolism [4, 64].

## Neuroinflammation

QUIN is responsible for metabolising more than 95% of L-tryptophan in the GI tract. QUIN facilitates the entry of L-tryptophan into the bloodstream and its passage through the blood–brain barrier (BBB), a critical factor for central serotonergic signalling. Consequently, the QUIN pathway is linked to neurodegenerative processes due to its essential role in the biosynthesis of several neuroactive intermediates, such as kynurenic acid, niacin, and NAD + . Additionally, QUIN is involved in the production of neurotoxic intermediates, promoting excessive stimulation of the NR2A and NR2B subunits of the NMDA receptor. This excessive stimulation leads to increased calcium influx into neurons, contributing to the generation of reactive oxygen species (ROS) and free radicals [52]. This process ultimately results in neuronal damage and death through mechanisms such as lipid peroxidation, which compromises membrane fluidity and permeability [82, 96].

Experimental evidence demonstrates that QUIN impairs blood–brain barrier (BBB) function by inducing the production of nitric oxide. This nitric oxide production triggers the hyperphosphorylation of cytoskeletal intermediate protein filaments in astrocytes and neurons, leading to significant cellular and molecular disruptions. These disruptions contribute to increased BBB permeability and promote neuroinflammatory processes [12]. The effects of QUIN are particularly pronounced in brain regions with high neuronal susceptibility, such as the cortex, striatum, and hippocampus, which are commonly affected in neurodegenerative diseases like Alzheimer's disease (AD). At low concentrations, QUIN can induce stem cell proliferation and serves as an intermediate in the synthesis of NAD + in human brain cells. However, the damage induced by QUIN varies depending on the brain region, with cortical, striatal, and hippocampal neurons being especially sensitive. This variability in neuronal sensitivity may partly explain the elevated levels of neurodegeneration observed in these regions in AD patients, which correlates with increased QUIN levels and associated inflammatory processes [83].

3-HK also induces oxidative stress and neuronal apoptosis through its interaction with xanthine oxidase, leading to the production of ROS such as superoxide radicals (O2-) and hydroxyl radicals (OH-) [84]. These ROS can cleave DNA and promote apoptosis, resulting in neuronal damage and cognitive and motor dysfunctions. Both L-kynurenine and 3-HK exhibit similar physiological characteristics and distribution within the CNS, with higher concentrations observed in the cerebral cortex, striatum, and hippocampus ([120]). Despite the relatively low number of *in vivo* studies, high levels of 3-HK have been associated with neuroinflammation, demonstrating both antioxidant and pro-oxidant properties depending on concentration. Low concentrations of 3-HK are linked to strong pro-oxidant activity and neuronal toxicity, whereas higher concentrations increase resistance to oxidation [47].

The physiological balance between neurotoxic kynurenines, such as 3-HK and QUIN, and neuroprotective kynurenines, such as KYNA, is crucial for CNS homeostasis, contributing significantly to neuroprotection against oxidative stress and ROS production [23]. As previously mentioned, under normal physiological conditions, astrocytic QUIN produces KYNA, a neuroprotective agent, while neuronal QUIN synthesises NAD + , thereby improving cellular energy status. In contrast, under pathological conditions, inflammatory signals stimulate the QUIN pathway in macrophages, microglia, and dendritic cells, leading to the production of high amounts of QUIN. This necessitates a regulatory balance controlled by IDO, which activates L-tryptophan catabolism via the QUIN pathway instead of the alternative serotonin production pathway. Inflammation triggers the production of metabolites like 3-HK and QUIN, affecting cognitive function and promoting neurodegeneration [101].

The imbalance among neurotoxic, neuroprotective, and immunomodulatory QUIN metabolites is reported in several neurodegenerative diseases, including Alzheimer’s disease (AD), Parkinson’s disease (PD), multiple sclerosis (MS), amyotrophic lateral sclerosis (ALS), and Huntington’s disease (HD). The KP is critical in these conditions due to its role in tryptophan metabolism and the production of neuroactive compounds like serotonin, KYN, and KYNA, which significantly impact brain function and fatigue perception. In AD, disturbances in the KP result in elevated levels of neurotoxic QUIN and reduced levels of neuroprotective KYNA, contributing to neuroinflammation and the formation of amyloid plaques and tau tangles, hallmark features of AD pathology [59]. Similarly, in PD, KP dysregulation leads to increased levels of 3-HK and QUIN, inducing oxidative stress and mitochondrial dysfunction, which contribute to neuronal death and motor dysfunction. In MS, elevated levels of QUIN during active disease phases contribute to the inflammatory milieu and neuronal damage observed in MS lesions, exacerbating disease progression [38]. ALS, marked by the progressive degeneration of motor neurons, shows KP dysregulation with elevated levels of QUIN associated with excitotoxicity and oxidative stress, leading to motor neuron death [38]. Lastly, in HD, increased production of neurotoxic metabolites like QUIN results in neuronal loss and characteristic motor and cognitive dysfunctions, with elevated QUIN levels correlating with disease severity and progression [38].

The KP's involvement in neuroinflammation and its impact on neurotransmitter systems, including glutamatergic, GABAergic, dopaminergic, and noradrenergic neurotransmissions, underscores its importance in brain function and the pathogenesis of disorders such as depression and neurodegeneration [48, 81, 114].

## Gut Permeability and Neuroinflammation

The immune system, co-evolving with commensal microorganisms, has established a relationship characterised by mutualism and homeostasis. Effective host immunity prevents commensal microbes from overexploiting resources while maintaining tolerance to harmless stimuli [1], [117]. Disruptions induced by antibiotics, dietary changes, or environmental pollutants can destabilise the gut microbiome ([18]). This destabilisation may impair the interfaces between host and microbiome, altering immune responses and potentially leading to systemic spread of commensal microbes, increased susceptibility to pathogenic invasion, and inappropriate immune reactions [25, 42].

The microbiota employs various defence mechanisms against colonisation, pathogen overgrowth, and resultant damage or infection. One such mechanism is colonisation resistance, where both commensal and pathogenic microorganisms compete for resources and functional space, often mediated by quorum sensing [42], [123, 124]. Integral to this defence are the intestinal epithelial cells (IECs), primarily enterocytes, which form the gut lining and regulate the trans-epithelial movement of substances. This lining is reinforced by a complex array of junction proteins, including tight junctions (TJPs), adherens junctions, gap junctions, and desmosomes, ensuring structural integrity and function [112].

**Fig. 2:**
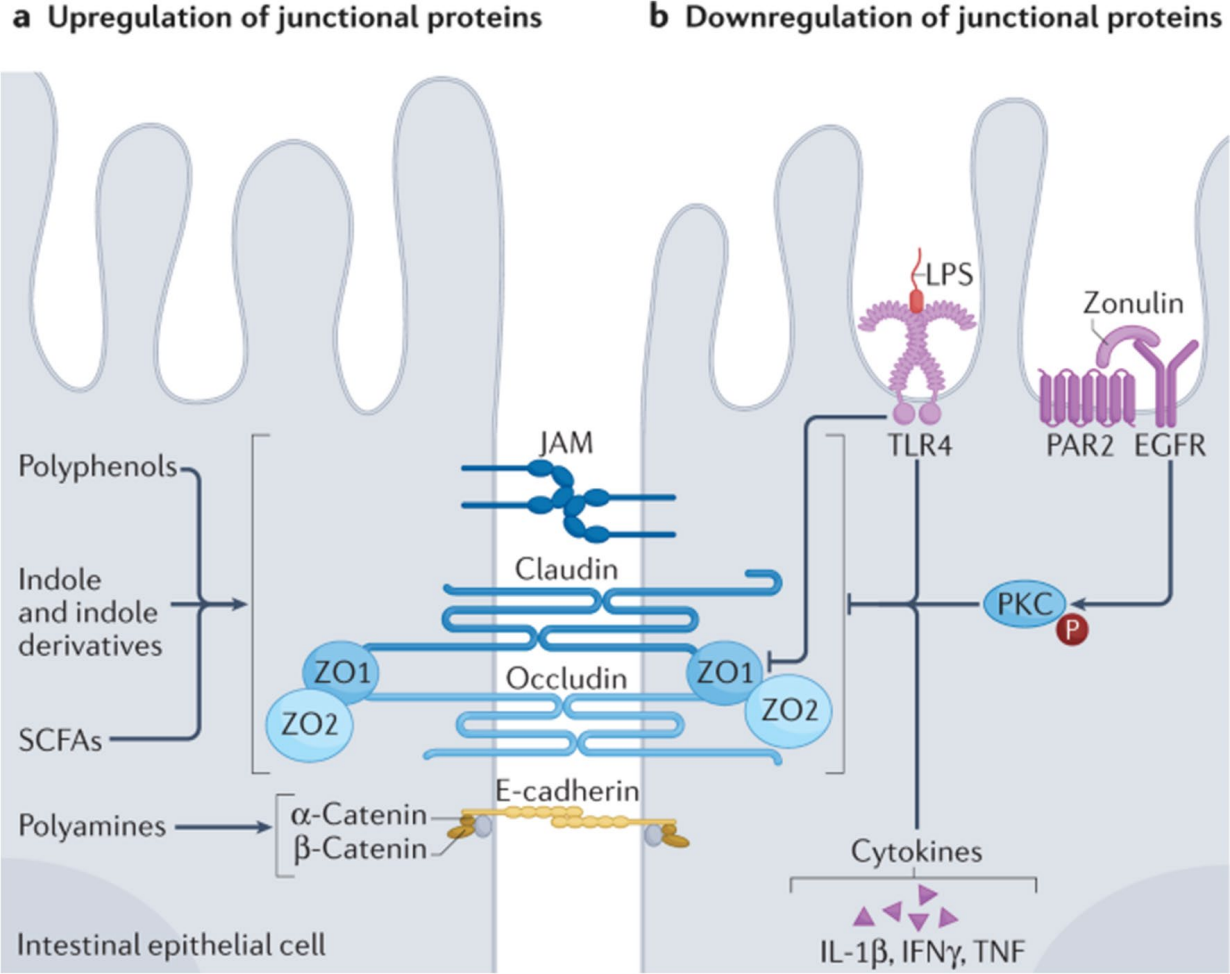
The gut epithelial barrier consists of the apical plasma membrane of enterocytes, held together by tight junction proteins (claudin and occludin) and adherens junction proteins (E-cadherin and catenin), as well as the zonula occludens proteins ZO1 and ZO2, which are adaptor proteins necessary for the structural and regulatory functions of tight junctions. **a** | Upregulation of junctional proteins can be induced by microbiota metabolites including polyphenols, indole and indole derivatives, short-chain fatty acids (SCFAs) and polyamines. **b** | Downregulation of junctional proteins is mediated by: lipopolysaccharides (LPS) through binding to Toll-like receptor 4 (TLR4); by zonulin, a protein that activates the EGF receptor (EGFR) through transactivation of the proteinase-activated receptor 2 (PAR2), thereby inducing protein kinase C (PKC) phosphorylation; and by pro-inflammatory cytokines, including IL-1β, interferon-γ (IFNγ) and tumour necrosis factor (TNF). JAM, junctional adhesion molecules.

The epithelial barrier (Fig. 2), functioning as a selectively permeable membrane, is regulated via two primary pathways: paracellular and transcellular transport. Paracellular transport allows the passive flow of ions and small molecules between epithelial cells, controlled by tight junctions involving proteins such as claudins, occludins, and junctional adhesion molecules [80]. In contrast, the transcellular pathway involves the active transport of substances across cell membranes, mediated by various transporters and channels, and requiring energy [91]. IECs, along with a protective mucus barrier, are pivotal in controlling the passage of substances. Tight junction proteins such as claudins, occludins, and ZO-1 regulate this permeability ([71]. IECs include various cell types: goblet cells produce mucus essential for maintaining the mucus barrier,enteroendocrine cells release hormones in response to luminal signals; sensory tuft cells are involved in immune responses; and Paneth cells contribute to mucosal defence through the secretion of antimicrobial peptides [29, 50, 61].

The human gut harbours a high concentration of gram-negative bacteria containing endotoxin, particularly in the lower intestine. These bacteria are also found in saliva, dental plaque, skin, lungs, respiratory tract, and urinary tract. Humans are highly sensitive to endotoxin, with significantly lower tolerance levels compared to other mammals. Recent studies have highlighted the role of tight junctions in maintaining epithelial permeability and their regulation by gut microbiota interactions. Dysbiosis, characterised by an imbalance in the gut microbial community, often leads to augmented intestinal permeability and subsequent chronic inflammation. This dysregulation is implicated in various diseases, including inflammatory bowel disease (IBD), metabolic disorders, and neurological conditions [3, 89, 121]. Furthermore, research has shown that endotoxin can significantly impact the kynurenine pathway, influencing neurological functions and contributing to conditions like Alzheimer's disease and Parkinson's disease [38, 79, 93].

Disruptions in the functionality of tight junctions or transporter activities can lead to increased epithelial permeability, allowing luminal antigens, pathogens, and toxins to translocate into the submucosa and systemic circulation. Such alterations can trigger immune responses and inflammatory cascades. GI barrier dysfunction enables endotoxins, such as LPS from gram-negative bacteria, to pass through the intestinal barrier into systemic circulation, a process termed endotoxemia. This passage of LPS can significantly impact the kynurenine pathway and subsequently influence neurological conditions [43]. LPS, consisting of lipid A, a short sugar chain core, and an O-antigen, is a major component of the outer membrane of gram-negative bacteria. Soluble endotoxin is released when bacteria are destroyed or physiologically as outer membrane vesicles. Different species of gram-negative bacteria have varying endotoxin structures, mainly due to differences in the O-antigen and lipid A, which are detected by the MD2/TLR4 receptor complex, determining the inflammation and toxicity of the endotoxin (Brown, 2019,Bryant et al., 2010).

Endotoxemia activates the local immune response, leading to the secretion of soluble factors such as soluble CD14 (sCD14) via the activation of T-lymphocytes, monocytes, and tissue macrophages. LPS binds to serum lipopolysaccharide-binding protein (LBP) to form an LPS-LBP complex, which then binds to the CD14 receptor on immune cells, initiating the production and release of pro-inflammatory cytokines such as Tumour Necrosis Factor Alpha (TNF-α) and Interleukin-6 (IL-6). This response disrupts GI barrier tight junction proteins, exacerbating intestinal permeability [43, 113].

The inflammatory response induced by endotoxin primarily involves the activation of Toll-like receptor 4 (TLR4) with its co-receptor MD2 on immune cells. This interaction initiates intracellular signalling cascades involving MyD88, TRAF6, and the IκB kinase (IKK) complex, leading to the activation of nuclear factor kappa-light-chain-enhancer of activated B cells (NF-κB). NF-κB translocates into the nucleus, upregulating genes encoding pro-inflammatory cytokines such as IL-6 and TNF-α [49, 88, 111]. IL-6 and TNF-α exert their effects by binding to their respective receptors, IL-6R and TNFR1/2, on target cells. IL-6 primarily activates the Janus tyrosine kinase and signal transducers and activators of transcription (JAK-STAT) pathway, leading to the phosphorylation and activation of STAT3, which regulates inflammatory gene transcription. TNF-α activates various signalling pathways, including NF-κB, Jun N-terminal kinase (JNK), and Mitogen-activated protein kinases (MAPK) pathways. Additionally, interferon-gamma (IFN-γ), another pro-inflammatory cytokine, upregulates the enzyme IDO, catalysing the conversion of tryptophan into N-formylkynurenine, initiating the KP. KMO further converts kynurenine into 3-hydroxykynurenine, an intermediate in the quinolinic acid pathway [64, 67]. Moreover, pro-inflammatory cytokines like IFN-γ can stimulate KMO, potentially promoting neurotoxicity by favouring the quinolinic acid pathway [52]. These inflammatory signals can affect neural drive, contributing to feelings of sadness and increased perceptions of fatigue, potentially leading to neuroinflammatory and neuropsychiatric conditions. Neuroinflammation mediated by the kynurenine pathway is implicated in the pathogenesis of various neurological disorders, including depression, Alzheimer's disease, and Parkinson's disease [6, 79, 115].

There are several mechanisms by which increased levels of cytokines in the periphery can reach and affect the brain. These include passage through leaky regions in the blood–brain barrier (BBB) such as circumventricular organs, active transport through transport molecules, activation of cells lining the cerebral vasculature (endothelial cells and perivascular macrophages), binding to cytokine receptors associated with the vagus nerve, stimulating the hypothalamic–pituitary–adrenal (HPA) axis at the anterior pituitary or hypothalamus, and recruitment of activated cells such as monocytes/macrophages from the periphery to the brain [10, 68, 69]. Through activation of the intracellular signalling pathway mitogen-activated protein kinase, cytokines can increase the number and function of the reuptake pumps for serotonin, noradrenaline, and dopamine, which in turn can reduce the availability of these neurotransmitters within the synaptic cleft. Preclinical studies have demonstrated that increased inflammatory cytokines reduce central levels of brain-derived neurotrophic factor (BDNF) and neurogenesis, leading to depressive-like behaviour [27]. However, the relationship between peripheral and central inflammatory markers and antidepressants is complex and it remains unclear which pathways are most relevant for cytokine signal transmission in stress-related disorders such as depression [10, 39], [56].

There is some evidence, albeit from small studies of short duration, suggesting that anti-inflammatory agents such as non-steroidal anti-inflammatory drugs (NSAIDs) and cytokine inhibitors reduce depressive symptoms [10]. For depressed patients with raised inflammatory markers, this raises the prospect of whether reducing low-grade inflammation could alleviate depressive symptoms. Although a randomised controlled trial of the monoclonal antibody infliximab, a TNF-α antagonist, was not superior to placebo in reducing depressive symptoms overall, in patients with high baseline CRP levels there were greater reductions in depressive symptoms than in those with low CRP levels [74]. Another study showed that CRP level at baseline differentially predicted treatment outcome with escitalopram or nortriptyline [41]. These studies provide the impetus for stratification of depressed patients based on inflammatory profiles to advance personalised medicine. Developing more nuanced profiles of inflammatory proteins and gene expression, as well as cellular immune parameters, likely represents the future for predictors and targets of response to anti-inflammatory therapies.

The brain regions most reliably identified as being most affected by the administration of inflammatory stimuli include the basal ganglia and the dorsal anterior cingulate cortex (dACC). The dACC, part of the brain’s limbic system, is involved in cognitive and emotional processing. Cytokines can induce increases in neural activity most strongly in either the subgenual or the dorsal area of the dACC and have been associated with the development of mood and anxiety symptoms ([56]). Cytokines can impair basal ganglia functioning through known inhibitory effects on dopamine signalling in the CNS [68]. Reductions in basal ganglia activity have been noted in more posterior regions, where they are associated with fatigue, and in more ventral regions (such as the nucleus accumbens), where they have been linked to the development of anhedonia [19, 40].

Microglia are central to the inflammatory process and a source of cytokines [16]. These phagocytic innate immune cells account for approximately 10% of cells in the brain and contribute to the plasticity of neural circuits by modulating synaptic architecture and function [35]. Microglial process motility can be modulated by glutamatergic and GABAergic neurotransmission. Preclinical studies have shown that acute stress results in microglia activation and increased levels of pro-inflammatory cytokines in areas such as the hippocampus and hypothalamus [26]. Most studies show increases in activated microglia in response to chronic stress. Preliminary changes in the microenvironment of the microglia may result in a susceptibility to a secondary inflammatory stimulus. This concept of microglia priming may be of relevance to depression, which often requires multiple environmental “hits” [107]. In an environmental two-hit rodent model in which the first experimental manipulation targeted pregnant dams, and the second manipulation was given to the resulting offspring, exposure to prenatal immune challenge and peripubertal stress synergistically induced pathological effects on adult behavioural functions and neurochemistry [44]. Thus, early-life stress primes microglia, leading to a potentiated response to subsequent stress. Interestingly, the microbiota regulates microglia maturation and function. Clinically, microglial activation in the prefrontal cortex (PFC), anterior cingulate cortex (ACC), and insula in medication-free depressed patients has been demonstrated using translocator protein density measured by distribution volume in a positron emission tomography study [28, 107].

## Gut-Brain Axis and Cognitive Function

The GBA plays a crucial role in mental and cognitive health, with mechanisms under extensive research (Berding, Vlckova, et al., 2021; [15, 31, 46, 75]). This axis comprises a bidirectional communication network between the CNS and ENS, integrating emotional and cognitive brain centres with intestinal functions [92, 110]. Alterations in the gut microbiota may influence the peripheral and CNS, potentially affecting brain function and cognitive processes [42, 85, 102], [126]. This communication involves both direct and indirect signalling through chemical transmitters, neuronal pathways, and the immune system [110]. Chemical signalling includes the production of neuroactive compounds by the gut microbiota, such as γ-aminobutyric acid (GABA, noradrenaline, dopamine, serotonin, and amino acids like tyramine and tryptophan (Berding, Vlckova, et al., 2021 [14, 64],). Microbial metabolites, notably short-chain fatty acids (SCFAs) such as butyrate, propionate, and acetate, are by-products of dietary fibre fermentation by intestinal microorganisms [77, 100]. These compounds traverse the portal circulation, influencing the host’s immune system, metabolism, and neuronal cells within the ENS and vagus nerve pathways [78, 100]. SCFAs have been demonstrated to affect CNS function by regulating neuroplasticity, gene expression, and immune responses, with butyrate notably modulating the expression of brain-derived neurotrophic factor (BDNF and attenuating depressive-like behaviours in animal models (Berding, Carbia, et al., 2021 [53],).

Neuronal pathways, particularly the vagus nerve, play a pivotal role in the GBA by conveying sensory signals from the gut to the CNS. This transmission involves the activation of mechanoreceptors and chemoreceptors responsive to various chemical stimuli [15]. The ENS, often described as the "second brain," contains an extensive neuronal network that regulates gut functions and is influenced by the gut microbiota, impacting gut motility and intestinal barrier function [5, 15]. Furthermore, the gut microbiota directly influences and is influenced by the immune system [42]. It plays a significant role in the development and function of the peripheral immune system and is integral to the healthy development, maturation, and activation of microglia, the innate immune cells in the brain ([18]). Signals from microbial metabolism are crucial for microglial function, as shown in studies where the restoration of microglial morphology and function in germ-free (GF) mice treated with bacterial-derived SCFAs was noted [35].

Additionally, the gut microbiota's interaction with the brain is mediated through the systemic immune system via circulating cytokines, which can alter immune signalling within the brain, potentially leading to symptoms such as loss of appetite, irritability, and low mood([20], [56, 95]). Research has also suggested that the gut microbiota influences the permeability of the BBB, with GF mice exhibiting increased BBB permeability partly due to reduced expression of tight-junction proteins such as occludin and claudin 5 [13].

## Gut Microbiota and Neuroimmune Pathways

The metabolism of the kynurenine pathway is intricately regulated by inflammatory mediators and immunoresponsive enzymes([17]). The gut microbiota plays a vital role in educating and regulating the host's immune system throughout life [34]. This regulatory function is evidenced not only in germ-free (GF) animals but also in those with depleted gut microbiota due to antibiotic treatment, which exhibit compromised immune responses to infections [54]. Conversely, the immune system shapes the composition and diversity of the intestinal microbiota [55].

GF animals display an immature immune system, potentially explaining the reduced kynurenine pathway metabolism observed in these animals [24]. Upon colonisation post-weaning, normal metabolic functions are restored, aligning with the reinstatement of immune system function following the introduction of intestinal microbiota [24, 90]. These findings have translational relevance as low-grade immune activation in IBS correlates with alterations in gut microbiota and increased KP metabolism [2]. The aryl hydrocarbon receptor, which responds to both exogenous and endogenous stimuli, modulates immune responses and maintains host-microbe homeostasis. Indole, produced from tryptophan by microbes, acts as a ligand for this receptor [9, 116]. Although kynurenine was traditionally seen as an inert precursor, it activates the aryl hydrocarbon receptor [58], which in turn regulates IDO and TDO expression [9, 60].

The complex interplay between the gut microbiota, kynurenine pathway metabolism, and the immune response is exemplified by increased kynurenic acid levels in the absence of aryl hydrocarbon receptors in mice and aryl hydrocarbon receptor activation in the brain following experimental stroke [9]. Additionally, astrocyte activity and CNS inflammation are modulated by type I interferons and tryptophan metabolites via the aryl hydrocarbon receptor, and administration of an aryl hydrocarbon receptor agonist can attenuate intestinal inflammation in mouse models of colitis ([21, 22]).

Microbial metabolites, such as SCFAs, also influence intestinal barrier integrity and systemic inflammation, leading to alterations in kynurenine pathway metabolism [108], [123, 124]. Notably, the gut microbiota regulates microglia maturation and function [35], yet KP metabolites in the CNS have not been reported in microbiota-deficient animals. Elevated levels of kynurenine and its metabolites have been observed in the brains of Toxoplasma gondii-infected mice, with reactivation linked to brain IDO activation via IFN-γ dependent mechanisms [33], [118]

The relationship between tryptophan metabolism and gut microbiota composition is supported by preclinical studies demonstrating increased circulating tryptophan levels in GF animals [24, 76]. Despite this, kynurenine pathway metabolism and circulating serotonin concentrations are decreased [24]. This aligns with findings that GI serotonin synthesis, influenced by microbial metabolites such as SCFAs or tryptophan-derived indole metabolites, modulates circulating levels [65, 92].

Infection with *Trichuris muris* increases the kynurenine/tryptophan ratio [97]. Preclinical studies highlight total tryptophan concentrations' role in brain uptake, although the dynamics of tryptophan flux down the KP warrant further investigation [83]. Increased circulating tryptophan levels in GF animals result in higher hippocampal serotonin concentrations [24]. However, it remains unclear whether reduced circulating kynurenine availability in microbiota-deficient animals affects CNS kynurenine and downstream metabolites.

Tryptophan metabolism via the KP has significant implications for neurogastroenterology due to its effects on GI and CNS functions and GBA signalling. IBS, characterised by altered tryptophan metabolism, is linked to GI symptoms and co-morbid mood and anxiety disorders [2, 51]. Mucosal kynurenic acid and 5-HT levels correlate with anxiety and depression scores in IBS patients [63]. Acute tryptophan depletion studies demonstrate the impact of peripheral tryptophan levels on CNS and ENS function, highlighting altered tryptophan metabolism in GBA dysregulation in IBS ([21, 22, 100]).

Mood and anxiety disorders are common in IBS, linked to inflammatory-mediated tryptophan metabolism along the KP ([21, 22, 92]). Dysregulated brain-gut communication impacts peripheral and central symptoms in IBS [51]. The GBA plays a crucial role in mental and cognitive health, integrating emotional and cognitive brain centres with intestinal functions through direct and indirect signalling pathways [5, 15]. Microbial metabolites like SCFAs influence CNS function, regulating neuroplasticity and gene expression, while the gut microbiota affects BBB permeability and immune signalling within the brain [94].

## The Role of Probiotics in Regulating Inflammation, Cognitive Development, and the Kynurenine Pathway

Probiotics are living, non-pathogenic bacteria and yeasts that, when administered in adequate amounts, confer health benefits by promoting microbial balance, particularly in the digestive system [62]. They primarily include *Lactobacillus* and *Bifidobacterium* species or *Saccharomyces boulardii* [70]. These probiotic strains engage in various physiological activities, such as reducing the pH of the intestine, cell-to-cell signalling, inhibiting the colonisation of pathogenic microbes, and regulating the host's immune response [87]. A distinct category of probiotics known as "psychobiotics" has been identified for their potential to improve psychological and mental health, affecting mood, anxiety, focus, memory, and cognition [32, 105].

The gut microbiota (GM), comprising a complex community of microbes, their genomes, and metabolic products, plays an important role in maintaining host health [86]. The dominant bacterial phyla in the GM include *Bacteroidetes, Proteobacteria*, and *Actinobacteria*, with common genera being *Streptococcus, Pseudomonas, Bacteroides, Fusobacteria, Clostridium*, and *Lactobacillus* [62]. These gut bacteria contribute to chronic inflammation and defence mechanisms, preserve the mucosal barrier, and assist in metabolism [70, 77]. The GM is also involved in producing GI hormones, short-chain fatty acids, vitamins, and medication absorption [45, 108].

Probiotics can modulate the composition of the GM and restore gut ecosystem balance, offering potential therapeutic approaches for cognitive deficits [70, 98]. For instance, a probiotic mixture containing *Lactobacillus acidophilus*, *L. rhamnosus*, and *Bifidobacteria longum* administered for three months improved *Bifidobacteria* and *Lactobacilli* levels and symptoms of autism [109]. Similarly, the supplementation of *Bifidobacterium breve* strain A1 facilitated hippocampal learning and memory in a Parkinson’s disease mouse model by recovering the expression of synaptophysin and postsynaptic density protein-95 [93].

Probiotics exert their beneficial effects through various mechanisms. They produce antioxidant enzymes (catalase, superoxide dismutase) and antioxidants (butyrate, folate, glutathione), and chelate metal ions, reducing oxidative stress [73, 99], [119]. Additionally, probiotics can inhibit TLR activation, reducing inflammatory responses, enhancing BBB integrity, and improving neurological functions ([119]). Probiotics also influence cognitive function by upregulating brain-derived neurotrophic factor (BDNF), increasing monoamine levels, and enhancing neuroplasticity, potentially ameliorating depression ([57, 73]).

Experimental support for the use of probiotics as therapeutic targets comes from various animal studies. For instance*, L. rhamnosus* JB-1 modulates GABA receptor expression, resulting in reduced anxiety-like symptoms by activating the vagus nerve [14]. Probiotics have also shown to decrease pro-inflammatory cytokines (e.g., IL-6, TNF-α) and increase anti-inflammatory cytokines (e.g., IL-10, TGF-β) in the brain, improving the gut barrier and reducing LPS levels in the bloodstream, which can mitigate neuroinflammation [80].

Probiotics have been found to modulate the KP, reducing neuroinflammation and promoting cognitive health [78]. As mentioned previously, under normal physiological conditions, TRP is metabolised by TDO, maintaining the KP in equilibrium. However, inflammatory factors increase the activity of IDO, leading to a higher production of QUIN, which can disrupt cognitive functions [100]. Probiotics can potentially modulate the KP by influencing TRP metabolism. For example, supplementation with specific strains like *Lactobacillus reuteri* and *Bifidobacterium infantis* has been shown to reduce the levels of neurotoxic metabolites, promoting neuroprotection. A study by Rudzki and colleagues demonstrated that the administration of probiotics altered the gut microbiota composition, resulting in decreased levels of QUIN and increased levels of KYNA in the brain, which is associated with improved cognitive function [103].

Additionally, combination probiotic therapy, such as *Bifidobacterium lactis*, *B. bifidum*, *Lactobacillus casei*, and *L. acidophilus* in aging mice, mitigates age-related disruption of the blood–brain barrier and intestinal barrier integrity, thereby reducing plasma and cerebral LPS and pro-inflammatory cytokines like IL-6, TNF-α, TLR4, and NF-κB translocation in the brain [36]. This improvement in microbial composition is accompanied by enhanced memory functions and reduced neuronal and synaptic injuries, as well as decreased microglia activation in the brain [36].

Clinical trials and observational studies are essential to evaluate the efficacy of probiotics in cognitive health. For instance, supplementation with *Lactiplantibacillus plantarum* OLL 2712 for 12 weeks reduced inflammation and improved memory in elderly adults [104]. Probiotics such as *Lactobacillus casei Shirota* have been shown to alleviate constipation and abdominal pain in Parkinson’s disease patients (Cassani et al., 2011). Probiotic supplementation has also improved verbal memory and cognitive performance in elderly subjects [66].

A meta-analysis by Zhu and colleagues ([125]) reported that probiotics significantly improved cognitive functions, particularly in mild cognitive impairment. Another meta-analysis suggested that probiotics could improve insulin resistance, lipid metabolism, and cognitive and GI health in patients with Alzheimer’s disease, mild cognitive impairment, and Parkinson’s disease [122]

While probiotics offer health benefits, side effects, albeit rare, may occur including systemic infections, GI side effects, and immune stimulation ([72]). Probiotic-induced d-lactic acidosis can occur in individuals with short bowel syndrome, leading to neurological symptoms such as memory loss and delirium [11].

To ensure safe probiotic use, microbiome profiling is recommended to identify factors affecting individual responses. Manufacturers should re-evaluate older strains for antibiotic resistance and disclose each probiotic strain’s antibiogram. Research into animal models is encouraged to detect potential long-term impacts of probiotics, particularly next-generation strains. Companies must monitor and report adverse events in compliance with regulatory regulations [62].

## Future Perspectives

### Integrating Gut Permeability, the Kynurenine Pathway, and Neuroinflammation

The GM actively communicates with the CNS through neural, endocrine, and immune pathways. Preclinical studies utilising germ-free animals and faecal microbiota transplantation have demonstrated the impact of GM on neuroinflammatory responses, providing insights into the potential therapeutic applications of GM modulation in neuroinflammatory diseases. Probiotics are live beneficial bacteria, while prebiotics are substances that selectively promote the growth of beneficial gut microbiota. Targeted administration of specific probiotics or prebiotics holds promise for modulating the GM and reducing neuroinflammation. Further research is needed to identify specific strains and combinations that effectively modulate neuroinflammatory processes. Postbiotics are the metabolic byproducts of probiotic bacteria, including short-chain fatty acids, antimicrobial peptides, and bioactive molecules. These postbiotics exhibit immunomodulatory and anti-inflammatory properties, offering potential therapeutic avenues for neuroinflammatory disorders. Understanding the mechanisms of action and developing strategies to enhance the production and delivery of beneficial postbiotics are essential areas for future investigation. Faecal microbiota transplantation, which involves transferring faecal material from a healthy donor to a recipient with a dysbiotic GM, has shown promising results in the treatment of various GI disorders and is now being explored as a potential therapy for neuroinflammatory diseases.

## Conclusion

The kynurenine pathway (KP) plays a pivotal role in gut permeability and inflammation, significantly impacting the gut-brain axis (GBA) and contributing to various neuropsychiatric disorders. Our review has elucidated the mechanisms by which alterations in gut microbiota composition can increase gut permeability, triggering systemic inflammation and neuroinflammation. The KP, which metabolises tryptophan into neuroactive and neurotoxic compounds, serves as a critical mediator in this process. We have highlighted the dual nature of kynurenine metabolites, balancing neurotoxic effects of compounds like quinolinic acid with the neuroprotective properties of kynurenic acid.

Probiotics emerge as promising therapeutic interventions, capable of modulating the KP and reducing neuroinflammation. Experimental data indicate that specific probiotic strains can shift the balance towards neuroprotective metabolites, thereby mitigating cognitive and emotional disturbances associated with increased gut permeability and systemic inflammation. These findings not only underscore the importance of maintaining a healthy gut microbiota but also open avenues for novel therapeutic strategies targeting the GBA.

Future research should focus on delineating the specific probiotic strains and combinations that are most effective in modulating the KP and reducing neuroinflammation. Additionally, the development of advanced analytical methods for measuring KP metabolites in clinical settings will enhance our understanding of their role in neuropsychiatric conditions. Integrating gut permeability, the kynurenine pathway, and neuroinflammation into a cohesive framework will provide deeper insights into the pathophysiology of neuropsychiatric disorders and guide the development of targeted interventions. Thus, the modulation of the gut microbiota holds great potential for therapeutic advancements in treating both gastrointestinal and neurological conditions, paving the way for improved mental and cognitive health.

## Data Availability

No datasets were generated or analysed during the current study.

## Abbreviations

GBA: Gut-Brain Axis
GI: Gastrointestinal
CNS: Central Nervous System
KP: Kynurenine Pathway
Trp: Tryptophan
IDO: Indoleamine 2,3-Dioxygenase
TDO: Tryptophan 2,3-Dioxygenase
N-fKYN: N-formylkynurenine
KYN: Kynurenine
KAT: Kynurenine Aminotransferase
KYNA: Kynurenic Acid
KYNU: Kynureninase
3-HK: 3-Hydroxykynurenine
3-HAA: 3-Hydroxyanthranilic Acid
QA: Quinolinic Acid
QPRT: Quinolinate Phosphoribosyltransferase
NAD +: Nicotinamide Adenine Dinucleotide
AA: Anthranilic Acid
XA: Xanthurenic Acid
ACMS: 2-Amino-3-carboxymuconate-6-semialdehyde
PIC: Picolinic Acid
BBB: Blood-Brain Barrier
ROS: Reactive Oxygen Species
NMDA: N-methyl-D-aspartate
SCFA: Short-Chain Fatty Acid
ENS: Enteric Nervous System
LPS: Lipopolysaccharide
TLR4: Toll-Like Receptor 4
TNF-α: Tumour Necrosis Factor Alpha
IL-6: Interleukin-6
sCD14: Soluble CD14
NF-κB: Nuclear Factor Kappa-light-chain-enhancer of Activated B Cells
JNK: Jun N-terminal Kinase
MAPK: Mitogen-Activated Protein Kinases
IFN-γ: Interferon-gamma
HPA: Hypothalamic-Pituitary-Adrenal
BDNF: Brain-Derived Neurotrophic Factor
NSAIDs: Non-Steroidal Anti-Inflammatory Drugs
CRP: C-Reactive Protein
GF: Germ-Free
GM: Gut Microbiota
PFC: Prefrontal Cortex
ACC: Anterior Cingulate Cortex
SCFAs: Short-Chain Fatty Acids
GABA: Gamma-Aminobutyric Acid
PI3K/Akt: Phosphoinositide 3-Kinase/Protein Kinase B
ERK: Extracellular Signal-Regulated Kinase
TGF-β: Transforming Growth Factor Beta
